# Baseline cardiac magnetic resonance imaging versus baseline endomyocardial biopsy for the prediction of left ventricular reverse remodeling and prognosis in response to therapy in patients with idiopathic dilated cardiomyopathy

**DOI:** 10.1007/s00380-013-0415-1

**Published:** 2013-10-04

**Authors:** Takeru Nabeta, Takayuki Inomata, Yuichiro Iida, Yuki Ikeda, Miwa Iwamoto, Shunsuke Ishii, Takanori Sato, Ichiro Watanabe, Takashi Naruke, Hisahito Shinagawa, Toshimi Koitabashi, Ichiro Takeuchi, Mototsugu Nishii, Yusuke Inoue, Tohru Izumi

**Affiliations:** 1 Department of Cardio-angiology, Kitasato University School of Medicine, 1-15-1 Kitasato, Minami-Ku, Sagamihara, Kanagawa 252-0374 Japan; 2 Department of Diagnostic Radiology, Kitasato University School of Medicine, Sagamihara, Japan

**Keywords:** Fibrosis, Magnetic resonance imaging, Biopsy, Dilated cardiomyopathy

## Abstract

Endomyocardial biopsy (EMB) and late gadolinium enhancement cardiovascular magnetic resonance (LGE-CMR) imaging performed at baseline are both used to evaluate the extent of myocardial fibrosis. However, no study has directly compared the effectiveness of these diagnostic tools in the prediction of left ventricular reverse remodeling (LVRR) and prognosis in response to therapy in patients with idiopathic dilated cardiomyopathy (IDCM). Seventy-five patients with newly diagnosed IDCM who were undergoing optimal therapy were assessed at baseline using LGE-CMR imaging and EMB; the former measured LGE area and the latter measured collagen volume fraction (CVF) as possible predictive indices of LVRR and cardiac event-free survival. Among all the baseline primary candidate factors with *P* < 0.2 as per univariate analysis, multivariate analysis indicated that only LGE area was an independent predictor of subsequent LVRR (*β* = 0.44; 95 % confidence interval (CI) 0.87–2.53; *P* < 0.001), as indicated by decreasing left ventricular end-systolic volume index over the 1-year follow-up. Kaplan–Meier curves indicated significantly lower cardiac event-free survival rates in patients with LGE at baseline than in patients without (*P* < 0.01). By contrast, there was no significant difference in prognosis between patients with CVF values above (severe fibrosis) and below (mild fibrosis) the median of 4.9 %. Cox proportional hazard analysis showed that LGE area was an independent predictor of subsequent cardiac events (hazard ratio 1.06; 95 % CI 1.02–1.10; *P* ≤ 0.01). The degree of myocardial fibrosis estimated by baseline LGE-CMR imaging, but not that estimated by baseline EMB, can predict LVRR and cardiac event-free survival in response to therapy in patients with newly diagnosed IDCM.

## Introduction

Left ventricular (LV) remodeling is a major pathogenic mechanism in the progression of idiopathic dilated cardiomyopathy (IDCM) and is a confirmed predictor of future cardiac events, including severe heart failure (HF) [1]. In some IDCM patients, particularly those receiving β-blockers [2] or cardiac resynchronization therapy (CRT) [3], the left ventricle undergoes volume reduction and normalization of shape with a concomitant improvement in pump function, a positive therapeutic response known as LV reverse remodeling (LVRR) [4]. The incidence and degree of LVRR were higher in IDCM patients who survived for at least 6 months during drug therapy than in patients who died or required transplantation, indicating that LVRR after therapy is correlated with a better prognosis [5, 6].

Myocardial fibrosis is a common feature of LV remodeling, and the increased ventricular stiffness associated with excess collagen accumulation can increase the risk of HF in patients with chronic cardiovascular diseases such as hypertension [7, 8]. Histologic evaluation by endomyocardial biopsy (EMB) was once the standard tool for the quantification of myocardial fibrosis [8], but EMB is now being supplanted by late gadolinium enhancement cardiovascular magnetic resonance (LGE-CMR) imaging as the diagnostic standard [9]. However, there have been no reports comparing the effectiveness of baseline EMB with that of baseline LGE-CMR imaging in estimating myocardial fibrosis for the prediction of LVRR and prognosis in response to therapy. To the best of our knowledge, this is the first report to directly compare the prognostic significance of EMB-derived collagen volume fraction (CVF) with LGE-CMR imaging-derived LGE area in patients with newly diagnosed IDCM undergoing therapy.

## Patients and methods

### Subjects

A total of 91 patients with newly diagnosed IDCM and an LV ejection fraction (LVEF) of <45 % on baseline echocardiography were referred to the Kitasato University Hospital between January 2007 and June 2012. Both LGE-CMR imaging and EMB were performed in these patients to determine the etiology of cardiomyopathy and the extent of fibrosis prior to treatment. Exclusion criteria were the presence of significant coronary artery disease (defined as the presence of >50 % luminal stenosis on coronary angiography or prior myocardial infarction), myocarditis, severe valvular heart disease, and/or chronic renal failure (estimated glomerular filtration rate <30 ml/min). Patients whose CMR images were of poor quality were also excluded. Six patients who underwent mitral valvoplasty and/or left ventriculectomy during the follow-up period were excluded from final analyses. Ten patients who were unable to be followed for >6 months were also excluded. A total of 75 patients were finally selected as the study subjects. Therapies for HF were in accordance with current guidelines [10] and were administered by experienced cardiologists. The study protocol was approved by our institution’s committee on human investigation, and written informed consent was obtained from all patients prior to study initiation.

### Clinical measurement and morphometric evaluation

Relevant clinical parameters derived from general laboratory analyses, electrocardiography, and echocardiography were recorded at baseline and approximately 1 year later (326 ± 102 days). Baseline data were collected with patients in a clinically stable condition. Transthoracic echocardiography was performed using an APLIO SSA-770A system (Toshiba, Tochigi, Japan), and repeated by the same experienced ultrasonographers whenever possible. M-mode images were obtained in the left parasternal long-axis view to measure the dimension of each chamber. LVEF and LV end-systolic volume index (LVESVI) were calculated by the modified Simpson method using biplanar images from apical views.

### LGE-CMR imaging

All LGE-CMR examinations were performed at baseline using a 1.5-T clinical scanner (Signa HDxt 1.5T; GE Healthcare, Milwaukee, WI, USA) with a maximum gradient strength of 33 mT/m and a slew rate of 120 mT/m/s. An eight-channel phased-array coil and vector electrocardiograph (ECG) were used for signal reception and cardiac gating, respectively. ECG-gated two-dimensional LGE images were acquired 10–15 min after the intravenous injection of 0.2 mmol/kg gadolinium using segmented inversion recovery fast gradient-echo sequences with the following parameters: echo time, 4.2 ms; repetition time, 8.0 ms; views per segment, 24; flip angle, 20°; inversion time, 150–220 ms; bandwidth, ±25 kHz; number of excitations, 1; in-plane resolution, 1.5 × 1.7 mm^2^; field of view, 340 mm × 340 mm; slice thickness, 8 mm; interslice gap, 8 mm; and four slices acquired in the LV short axis over two R–R intervals. The presence of LGE was determined by two experienced and independent observers blinded to patient outcome. The extent of LGE was expressed as “LGE area,” defined as an area showing a signal intensity of ≥5 standard deviations (SDs) above the mean of the remote reference myocardium. LGE area was quantified by semiautomatic planimetry on the short-axis contrast images using Ziostation 2 (Ziosoft, Tokyo, Japan).

### EMB

At least three EMB specimens were obtained at baseline from the posterior wall of the LV chamber. The interval between LGE-CMR imaging and EMB was <3 weeks. When required, tissue sections were stained with Masson’s trichrome to distinguish cardiomyocytes from connective tissue, and labeled with antibodies against CD3, CD68, and tenascin C to exclude infiltrative myocarditis [11]. Serial images of the tissue sections were analyzed using a projection microscope (Lumina Vision 3.3.2.0; Mitani, Fukui, Japan) to estimate the degree of myocardial fibrosis. CVF was calculated by averaging the total connective tissue area in 10 representative fields on sections containing no endocardium or blood vessels [12]. Histologic evaluation was performed by two well-trained pathologists blinded to patient identity or condition.

### Clinical observation and statistical analysis

Patients were divided into groups by the presence or absence of LGE on baseline LGE-CMR images (LGE+ and LGE− groups) and by the median value of CVF derived from baseline EMB (<median CVF or mild fibrosis group, and ≥median CVF or severe fibrosis group). Continuous variables expressed as mean ± SD were compared between groups using Student’s *t* tests, whereas binary variables were compared using the Chi-square test with Yates’ correction for continuity when necessary. Correlation between LGE area and CVF were examined using Spearman’s rank-correlation test.

The change in LVESVI at 1 year after treatment as revealed by echocardiography was set as a primary end point of this study, considering the contributing factors for LVRR. To predict the extent of LVRR from baseline variables, univariate screening of all baseline clinical and laboratory variables was performed. Then a stepwise backward conditional algorithm was subsequently applied to selected candidates with *P* < 0.2 as per univariate analysis to estimate the multivariable regression equation. Event-free survival curves were drawn according to the Kaplan–Meier method and compared using the log-rank test. Cardiovascular events, including sudden death, readmission for HF exacerbation, and major ventricular arrhythmias were considered as secondary end points. For the univariate analysis, we included potential covariates that affect HF prognosis, as per earlier reports [4, 13–18]. To test for independent predictors of cardiac events, clinical variables with *P* ≤ 0.04 as per univariate analysis were examined using multivariate analysis in a Cox proportional hazard model. All statistical analyses were performed using JMP Pro 9.0.2 (SAS Institute, Cary, NC, USA). All *P* values were two-sided, and *P* < 0.05 was considered statistically significant.

## Results

### Baseline characteristics in relation to LGE measured using LGE-CMR imaging and CVF measured using EMB

**Fig. 1:**
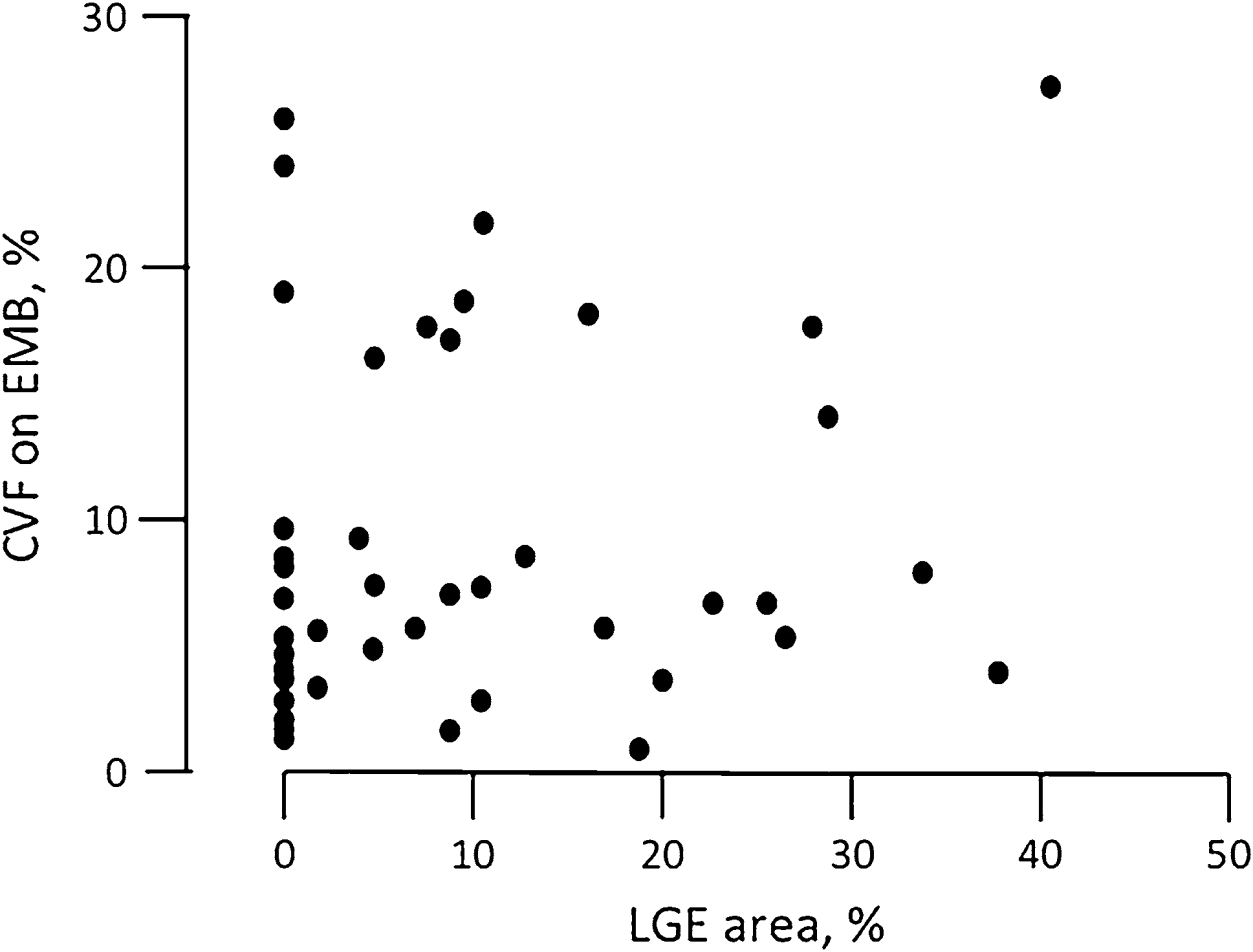
Correlation between late gadolinium enhancement (*LGE*) area as determined by LGE cardiac magnetic resonance images and collagen volume fraction (*CVF*) as determined by endomyocardial biopsy (*EMB*). There was no correlation between myocardial fibrosis estimated by LGE area and that estimated by CVF (*R* ^2^ = 0.182, *P* = 0.118)

In total, 75 patients (mean age 56 ± 13 years; 65 % male) admitted for treatment of IDCM met the inclusion criteria for this study. At baseline, LVEF was 30 ± 7 % while HF was sufficiently compensated in most cases, with 85 % of patients exhibiting New York Heart Association (NYHA) functional class ≤III under standard therapies. Thirty-six patients (48 %) exhibited positive LGE on LGE-CMR images, with a mean LGE area of 6.4 ± 9.9 %. There were no significant differences in clinical parameters between the LGE+ and LGE− groups, including CVF as determined by EMB, plasma B-type natriuretic peptide (BNP) and serum creatinine levels, QRS width, and LVESVI, with the exception of systolic blood pressure, which was higher in the LGE+ group (116 ± 14 mmHg) than in the LGE− group (108 ± 17 mmHg; *P* = 0.026; Table 1). Surprisingly, LGE area was not correlated with CVF (Fig. 1). Almost all patients were prescribed β-blockers together with angiotensin-converting enzyme inhibitors or angiotensin-II receptor blockers, while approximately half were also prescribed aldosterone blockers despite significant differences in carvedilol equivalent dose (10.0 ± 7.9 vs 15.0 ± 7.0 mg/day; *P* = 0.019). In addition, there were no significant differences in the proportion of patients with implantable defibrillation or CRT devices between the LGE+ and LGE− groups (Table 1).

**Table 1:** Baseline characteristics

	Total (*n* = 75)	LGE+ (*n* = 36)	LGE− (*n* = 39)	*P* value
Age (years)	56 ± 13	59 ± 14	54 ± 12	0.091
Sex, males, *n* (%)	49 (65)	24 (66)	25 (64)	0.816
NYHA (I:II:III:IV)	13:51:11:0	6:24:6:0	7:27:5:0	0.894
SBP (mmHg)	112 ± 16	108 ± 17	116 ± 14	0.026*
Heart rate (beats/min)	77 ± 13	75 ± 12	79 ± 13	0.143
Electrocardiographic data				
QRS duration (ms)	115 ± 26	113 ± 22	118 ± 29	0.388
Left bundle branch block, *n* (%)	10 (13)	1 (3)	9 (23)	0.015*
Echocardiogram data				
LVEF (%)	30.2 ± 7.3	31.4 ± 7.4	29.1 ± 7.1	0.163
LVESVI (ml/m^2^)	119 ± 36	126 ± 37	113 ± 35	0.106
Laboratory data				
BNP (pg/ml)	240 ± 196	236 ± 180	244 ± 212	0.903
BUN (mg/dl)	18.6 ± 6.16	19.4 ± 5.5	17.9 ± 6.67	0.288
Creatinine (mg/dl)	0.88 ± 0.25	0.85 ± 0.23	0.90 ± 0.28	0.373
Total bilirubin (mg/dl)	0.69 ± 0.30	0.73 ± 0.26	0.66 ± 0.34	0.326
Treatment, *n* (%)				
β-Blocker	71 (95)	33 (92)	38 (97)	0.345
Carvedilol equivalent dose (mg/day)	12 ± 7.6	10 ± 7.9	15 ± 7.0	0.019*
ACEI/ARB	74 (99)	36 (100)	38 (97)	1
MRB	46 (61)	26 (72)	20 (51)	0.096
ICD	6 (8)	4 (11)	2 (5)	0.337
CRT	5 (7)	3 (8)	2 (5)	0.578
CVF on EMB (%)	7.05 ± 6.24	6.52 ± 5.98	7.61 ± 6.54	0.432

Data given as mean ± SD or *n* (%)

*LGE* late gadolinium enhancement-cardiovascular magnetic resonance, *SBP* systolic blood pressure, *ACEI* angiotensin-converting enzyme inhibitor, *ARB* angiotensin receptor blocker, *MRB* mineralocorticoid receptor blocker, *ICD* implantable cardioverter-defibrillator, *CRT* cardiac resynchronization therapy, *BNP* brain natriuretic peptide, *BUN* blood urea nitrogen, *LVEF* left ventricular ejection fraction, *LVESVI* left ventricular end-systolic volume index, *CVF on EMB* collagen volume fraction on endomyocardial biopsy

* *P* < 0.05

### Baseline LGE and CVF as predictors of LVRR and prognosis

**Fig. 2:**
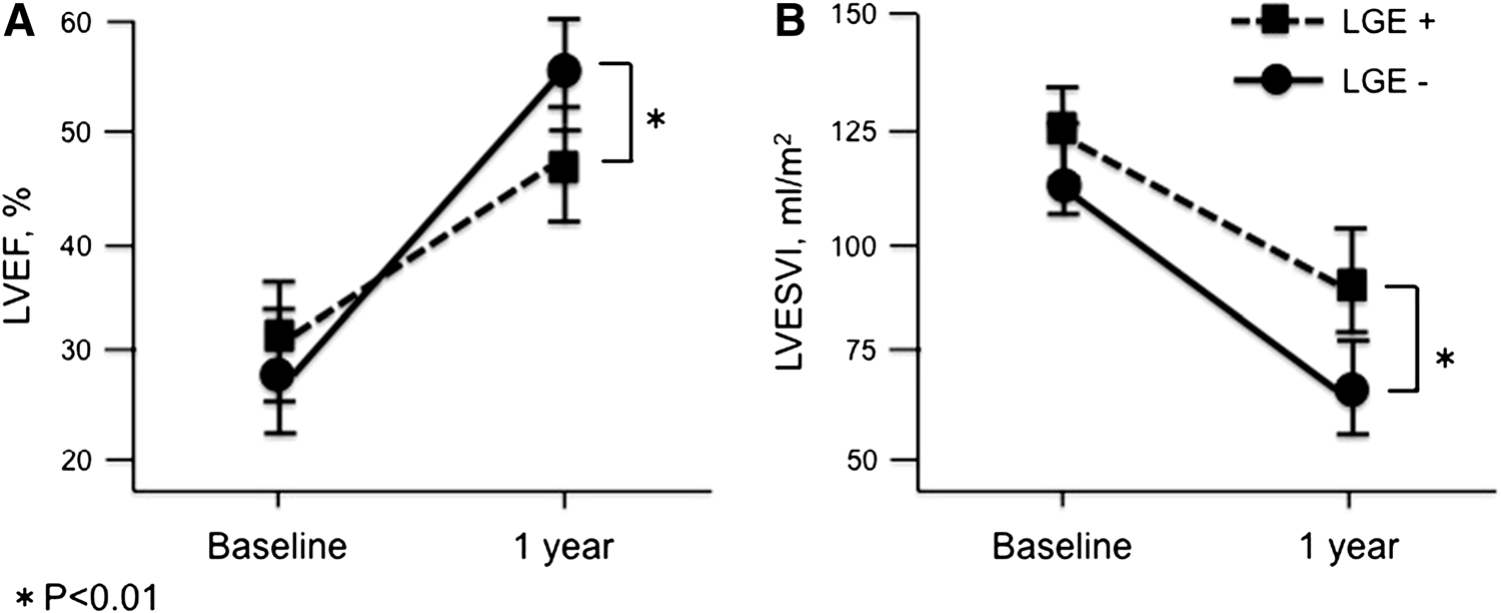
Morphometric and functional changes in the left ventricle as evaluated by echocardiography in patients with or without late gadolinium enhancement (*LGE*) determined by LGE cardiac magnetic resonance (LGE-CMR) imaging at baseline The relationship between baseline LGE (LGE+ or LGE−) and LV ejection fraction (*LVEF*; **a**) and LV end-systolic volume index (*LVESVI*; **b**) in response to 1 year of therapy

Morphometric and functional deterioration of the left ventricle was evaluated by echocardiography. Baseline characteristics were similar between groups defined by the presence of LGE and by the CVF value. However, LVRR at approximately 1 year after the initiation of optimal therapy was more advanced in the LGE− group than in the LGE+ group (Fig. 2). Univariate analysis showed that the significant predictors of subsequent LVRR among the different clinical parameters at baseline included LGE area, but not CVF. Of all the primary candidate factors with *P* < 0.2 as per univariate analysis, multivariate analysis indicated that only LGE area (*β* = 0.44; 95 % confidence interval (CI) 0.87 − 2.53; *P* < 0.001) and plasma BNP levels were independent predictors of subsequent LVRR (Table 2).

**Table 2:** Univariate and multivariate analyses for the change of LVESVI

	Univariate analysis			Multivariate analysis		
	*β* coefficient	95 % CI	*P* value	*β* coefficient	95 % CI	*P* value
Age	−0.02	−0.74 to 0.62	0.862			
SBP	−0.057	−0.71 to 0.43	0.625			
Heart rate	−0.067	−0.92 to 0.51	0.567			
QRS duration	−0.032	−0.40 to 0.31	0.787			
Left bundle branch block	−0.098	−18.7 to 7.60	0.404			
BNP	−0.153	−0.07 to 0.02	0.189	−0.24	−0.09 to −0.01	0.028*
BUN	−0.089	−2.02 to 0.90	0.447			
Creatinine	−0.024	−39.2 to 31.8	0.836			
Total bilirubin	−0.034	−18.7 to 7.60	0.404			
β-Blocker dose	−0.091	−1.64 to 0.72	0.436			
CVF on EMB	0.038	−1.19 to 1.65	0.333			
LGE area	0.388	0.67 to 2.35	<0.001**	0.436	0.87 to 2.53	<0.001**

*β*-Blocker dose expressed as equivalent to carvedilol

*SBP* systolic blood pressure, *BNP* brain natriuretic peptide, *BUN* blood urea nitrogen, *CVF on EMB* collagen volume fraction on endomyocardial biopsy, *LGE* late gadolinium enhancement cardiovascular magnetic resonance

* *P* < 0.05, ** *P* < 0.01

**Fig. 3:**
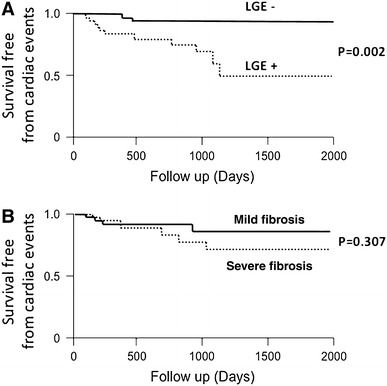
Event-free survival in groups stratified by late gadolinium enhancement (*LGE*) determined by LGE cardiac magnetic resonance imaging, and collagen volume fraction (*CVF*) determined by endomyocardial biopsy. Kaplan–Meier analysis illustrates poorer long-term outcome in patients with LGE positivity (LGE+) than in patients with LGE negativity (LGE−) at baseline (**a**). By contrast, no difference in long-term outcome was found between the group with CVF below the median value (mild fibrosis) and the group with CVF above the median value (severe fibrosis) (**b**)

The cardiac events were observed in 13 patients (11 LGE+ and 2 LGE− cases): 11 with HF hospitalization and 2 with major ventricular arrhythmias. Kaplan–Meier curves indicated significantly higher cardiac event-free survival rates in the LGE− group than in the LGE+ group (*P* < 0.01), but there was no significant difference in prognosis between the group with a CVF value above the median (4.9 %) and the group with a CVF value below the median (Fig. 3). Univariate Cox proportional hazard analysis showed that the significant predictors of subsequent cardiac events (*P* ≤ 0.04) among the different clinical parameters at baseline included LGE area and systolic blood pressure. Multivariate analysis indicated that out of these primary candidates, only LGE area was an independent predictor of subsequent cardiac events (hazard ratio 1.06; 95 % CI 1.02–1.10; *P* ≤ 0.01; Table 3).

**Table 3:** Cox proportional hazard regression analysis for incidence of cardiac events

	Univariate analysis			Multivariate analysis		
	Hazard ratio	95 % CI	*P* value	Hazard ratio	95 % CI	*P* value
Age	1.013	0.97–1.06	0.53			
Male	0.597	0.20–1.86	0.361			
SBP	0.958	0.92–1.00	0.026*	0.986	0.94–1.02	0.466
Heart rate	0.979	0.94–1.02	0.339			
Left bundle branch block	0.006	0.99–1.00	0.049			
BNP	1.002	0.99–1.00	0.075			
LVEF	1.072	0.99–1.16	0.069			
LVESVI	1.002	0.99–1.02	0.748			
β-Blocker dose	0.931	0.86–1.00	0.055			
CVF on EMB	1.055	0.98–1.13	0.159			
LGE area	1.068	1.03–1.11	<0.001**	1.06	1.02–1.10	0.009**

*β*-Blocker dose expressed as equivalent to carvedilol

*SBP* systolic blood pressure, *BNP* brain natriuretic peptide, *BUN* blood urea nitrogen, *LVEF* left ventricular ejection fraction, *LVESVI* left ventricular end-systolic volume index, *LGE* late gadolinium enhancement-cardiovascular magnetic resonance, *CVF on EMB* collagen volume fraction on endomyocardial biopsy

* *P* < 0.04, ** *P* < 0.01

## Discussion

### Mechanism and clinical relevance of LGE-CMR

CMR imaging is now established as the reference imaging method to assess cardiac anatomy and function together with myocardial characterization [19]. In particular, LGE-CMR imaging can detect the presence and extent of myocardial fibrosis [9]. An earlier study that involved the examination of myocardial tissue samples obtained from autopsy or heart transplantation revealed segmental and replacement fibrosis corresponding to LGE-CMR findings [20]. The presence of LGE has also been associated with a marked increase in LV volume and severely impaired systolic function [21]. Furthermore, several reports have demonstrated that LGE-CMR imaging is a predictor of adverse outcome in patients with IDCM [22, 23], as shown in this study. The overall occurrence of LGE (48 %) at baseline in this patient cohort with confirmed IDCM was similar to that found in previous studies [22, 23] (Table 1), indicating that the methodology was sufficiently sensitive to detect fibrosis associated with IDCM.

### Clinical parameters predictive of LVRR

The presence and extent of LVRR is of potential prognostic value for the stratification of long-term risk in patients with impaired LV contraction [24]. Although the precise mechanisms of LVRR have not been elucidated, several hypothetical mechanisms have been proposed on the basis of the efficacy of specific clinical interventions. These include hemodynamic improvement, direct action by cardiomyocytes, and electrical/mechanical resynchronization [25]. A few previous studies have attempted to identify the early clinical characteristics of IDCM that are predictive of LVRR and useful for long-term prognosis in response to tailored medical therapy. Merlo et al. [26] found that higher baseline systolic blood pressure and the absence of left bundle branch block were predictors of LVRR in a large population of IDCM patients. Kawai et al. [27] found that higher systolic blood pressure and lower pulmonary arterial wedge pressure at diagnosis were independent predictors of LVRR. The present study, however, did not show an association between any hemodynamic parameters and the presence or extent of LVRR, presumably because this study included a distinct clinical group composed exclusively of newly developed IDCM patients in a clinically stable condition.

Several cardiac imaging modalities have been developed to predict subsequent LVRR. Several studies have suggested that iodine-123 metaiodobenzylguanidine (MIBG) myocardial scintigraphy is a particularly powerful tool, not only for detecting myocardial abnormalities in the adrenergic nervous system of IDCM patients [28] but also for predicting the response to β-blocker therapy [29]. However, MIBG scintigraphy has not yet achieved broad clinical acceptance for these purposes because the quantitative parameters differ between institutions and the tracer is not widely available [30]. On the other hand, CMR provides highly accurate and reproducible measures of pathologic tissue changes, including myocardial fibrosis. The degree of myocardial fibrosis detected by baseline LGE-CMR imaging in this study was an independent predictor of LVRR in response to optimal medical therapy (Table 2; Fig. 2), consistent with the findings of previous reports [31].

### Baseline LGE-CMR imaging versus EMB as indicators for myocardial fibrosis

Myocardial fibrosis is associated with both ventricular remodeling leading to HF and a scar-related re-entrant mechanism linked to ventricular arrhythmia. Although it has been established that both LGE and CVF as measured by LGE-CMR imaging and EMB can estimate the extent of fibrosis in myocardial tissue [20], there was no significant correlation between LGE and CVF (Fig. 1) in this study. Mean CVF in the present study was 7.2 %, consistent with that reported in previous studies [32]; on the other hand, approximately half of the patients were LGE− (Table 1). The reasons for this apparent discrepancy remain to be explained, but may relate to the pathogenesis of myocardial fibrosis. Several mechanisms are believed to contribute to the development of myocardial fibrosis, such as inflammation, neurohumoral changes, and microvascular ischemia [33]. Both reactive and reparative patterns of fibrosis are seen in patients with IDCM. One report found that the presence of focal fibrosis as detected by LGE-CMR imaging in patients with IDCM was related to reparative inflammation but not to reactive diffuse interstitial fibrosis [33]. Alternatively, diffuse cardiac fibrosis, mainly of reactive origin, cannot be detected by LGE-CMR imaging [34]. Therefore, a normal LGE-CMR imaging study does not exclude increased interstitial fibrosis. Scan sequences for LGE-CMR imaging of diffuse interstitial fibrosis, such as T1 mapping [35] and equilibrium contrast CMR [36], are still in the experimental stages.

### Baseline LGE-CMR imaging versus baseline EMB as predictors of LVRR and prognosis

A hallmark of myocardial damage in IDCM patients is myocardial fibrosis. However, the extent of myocardial fibrosis at first presentation and its association with subsequent responses to various therapies have not been examined in requisite detail. The present multivariate analysis clearly demonstrated the prognostic value of LGE area, but not that of CVF, as a predictor of poor LVRR (Table 2) and adverse outcome (Table 3; Fig. 3). The reasons for this result are undetermined. We suggest that LGE-CMR imaging may yield more information about fibrosis over the entire left ventricle in comparison with EMB. The imaging power of LGE-CMR is based on a combination of increased accumulation/distribution of the contrast agent and a prolonged washout period related to the decreased capillary density within myocardial fibrotic tissue [37]. In contrast to the present results, several studies have found a good correlation between CVF and both cardiac events [38] and LVRR [39] in response to β-blockers and other regimens, whereas others have not [26, 40]. However, EMB has several inherent limitations. First, sampling errors restrict the accuracy of biopsy in patients with localized fibrosis, and fibrotic involvement over the entire left ventricle cannot be determined [41]. Furthermore, the predictive value of a local sample depends on the uniformity of myocardial fibrosis, which in turn is related to etiology. CVF may be substantially higher or lower than the average value if fibrosis is highly localized. Although the underlying processes leading to focal fibrosis in IDCM patients are currently unknown, several reports have suggested that focal fibrosis reflects the transition from a compensated (reparative) to a decompensated state associated with a poor prognosis [13].

### Clinical implications

The findings of this study have important implications for the clinical management of patients with newly diagnosed IDCM. LGE positivity at baseline may signify the need for more aggressive therapies. The ability to estimate the likelihood of LVRR (or risk of poor LVRR) before initiating therapy should influence treatment strategy. LVEF is a major determinant of feasible therapeutic options, including device implantation, but baseline LVEF may be a poor predictor of subsequent LVRR and prognosis in patients with new-onset IDCM [26]. When the probability of LVRR is high, even if the baseline LVEF is low, it may be possible to delay dangerous or invasive therapeutic options and wait for subsequent improvement in LV function through pharmacotherapy. For example, although an LVEF of <35 % is a criterion for the use of implantable cardiac defibrillators and/or CRT, these costly and highly invasive treatments may not be necessary for patients with newly diagnosed IDCM and LGE negativity at baseline.

### Study limitations

The present study had some limitations. It was a single-center study, so selection bias was a major concern. Because of the low number of cardiac events in our cohort, which comprised a relatively small number of patients, the findings of this observational study should be interpreted with caution. Although LGE-CMR imaging allows for semiqualitative assessment of myocardial structure and function, it is of limited accuracy for the absolute quantification of myocardial fibrosis. Indeed, there is no clear consensus on the optimal intensity threshold to identify fibrosis. Reports have demonstrated the efficacy of LGE patterns such as mid-wall, epicardial, diffuse, and focal enhancement in patients with IDCM [22, 23]. However, the present sample size was insufficient to establish any correlation between different LGE patterns and LVRR in terms of differential risk.

## Conclusion

In patients with newly diagnosed IDCM, the degree of myocardial fibrosis detected by LGE-CMR imaging, but not that estimated by EMB, was an independent predictor of LVRR and prognosis in response to optimal medical therapy.
